# Versatile single-step-assembly CRISPR/Cas9 vectors for dual gRNA expression

**DOI:** 10.1371/journal.pone.0187236

**Published:** 2017-12-06

**Authors:** Fatwa Adikusuma, Chandran Pfitzner, Paul Quinton Thomas

**Affiliations:** 1 School of Biological Sciences, The University of Adelaide, Adelaide, South Australia, Australia; 2 Centre for Biomedical Research, Faculty of Medicine, Diponegoro University, Semarang, Central Java, Indonesia; 3 Robinson Research Institute, The University of Adelaide, Adelaide, South Australia, Australia; 4 South Australian Health and Medical Research Institute, Adelaide, South Australia, Australia; SRI International, UNITED STATES

## Abstract

CRISPR/Cas9 technology enables efficient, rapid and cost-effective targeted genomic modification in a wide variety of cellular contexts including cultured cells. Some applications such as generation of double knock-outs, large deletions and paired-nickase cleavage require simultaneous expression of two gRNAs. Although single plasmids that enable multiplex expression of gRNAs have been developed, these require multiple rounds of cloning and/or PCR for generation of the desired construct. Here, we describe a series of vectors that enable generation of customized dual-gRNA expression constructs via an easy one-step golden gate cloning reaction using two annealed oligonucleotide inserts with different overhangs. Through nucleofection of mouse embryonic stem cells, we demonstrate highly efficient cleavage of the target loci using the dual-guide plasmids, which are available as Cas9-nuclease or Cas9-nickase expression constructs, with or without selection markers. These vectors are a valuable addition to the CRISPR/Cas9 toolbox and will be made available to all interested researchers via the Addgene plasmid repository.

## Introduction

CRISPR/Cas9 technology is a powerful genome editing tool that has become widely used by researchers to generate targeted genetic modifications in many contexts including cultured cell lines and zygotes. CRISPR/Cas9 offers several advantages over preexisting genome editing technologies including ease of use, relatively low cost and high activity [1–5]. The CRIPSR/Cas9 platform comprises two components; Cas9, which functions as a programmable endonuclease that generates a blunt-ended double-stranded break (DSB) and a ~100 nt guide RNA (gRNA), in which the ~20 nt at the 5’ end directs Cas9 to the target site via RNA:DNA complementary base pairing [6–8]. Generation of a targeted DSB can be achieved by delivery of Cas9 and gRNA components in plasmid, RNA or ribonucleoprotein (RNP) forms. For some applications, such as cultured cells, plasmids are generally preferred due to their ease of generation and stability. Commonly used plasmids for expression of Cas9 or Cas9-nickase (D10A) and single gRNA are available from the Zhang laboratory and can be obtained through the Addgene plasmid repository. These plasmids contain both gRNA and Cas9 expression cassettes in a single plasmid with optional selection markers such as puromycin or GFP to facilitate screening. Importantly, generation of a unique customized gRNA of interest can be performed easily as the gRNA cloning site contains BbsI restriction sites, allowing a one-step golden gate cloning approach for insertion of a pair of annealed oligonucleotides containing the specific ~20 bp guide sequence [6, 9].

To simultaneously target a pair of genomic regions, expression of two gRNAs is required. While this can be achieved by co-transfection of two plasmids, this process can be inefficient. To achieve efficient dual cuts, all CRISPR/Cas9 components with dual-gRNAs should be expressed from a single plasmid. Single plasmids expressing multiple gRNAs have been developed, however generation of the desired constructs using those available plasmids require multiple cloning and/or PCR steps. Here we modify commonly-used vectors from the Zhang laboratory so that each plasmid can express two gRNAs and can be generated via a simple one-step cloning method. We show that these plasmids, termed dual-gRNA plasmids, provide an efficient tool for experiments requiring simultaneous expression of two gRNAs such as multiplexed knock-out of two genes, generation of large deletions and generation of indels using Cas9-nickase. These vectors are a valuable addition to the CRISPR/Cas toolbox and will be made available through the Addgene plasmid repository.

## Results

### Generation of vectors

**Fig 1:**
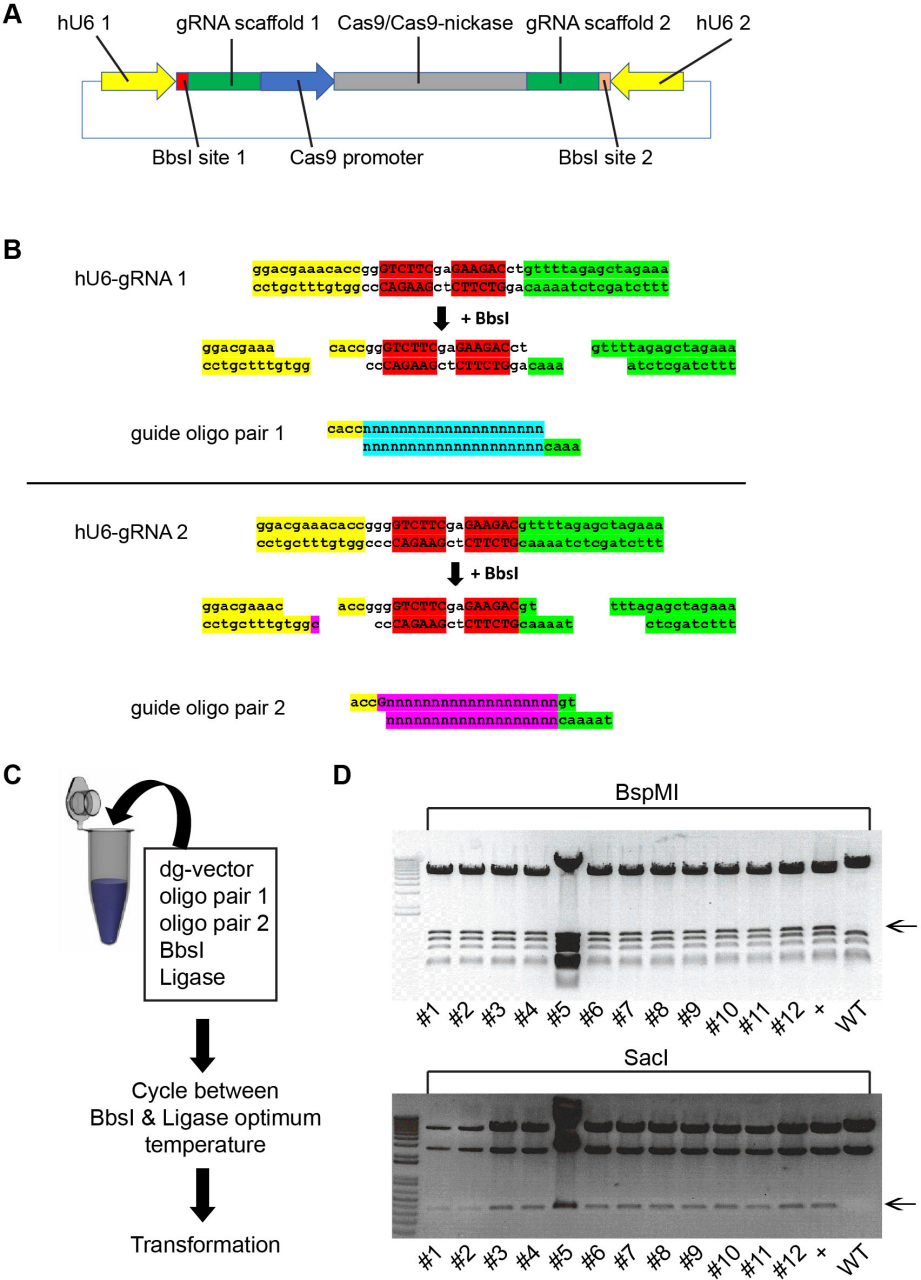
Generation of dual-gRNA expressing vectors. (A) Schematic of dual-gRNA vectors. (B) Golden gate cloning strategy for insertion of specific guide sequences into each cassette. Note that the BbsI sites generate different overhangs after restriction digest. Red highlights indicate the BbsI sites, yellow and green highlights are part of hU6 promoter and gRNA, respectively, that are necessarily present in the plasmid. Blue and purple highlights indicate the unique customized guide sequences (C) One-step cloning protocol for the generation of customized dual-gRNA vectors. (D) Insertion of Sox1A and Sox3A oligonucleotide duplexes into pDG459 resulted in correct insertions in all 12 colonies as indicated by BspMI and SacI restriction digest. The black arrow indicates the diagnostic band for correct insertion.

To generate plasmids that permit simultaneous expression of two gRNAs, we inserted an additional hU6-gRNA expression cassette into the available CRISPR plasmids from the Zhang laboratory. The second cassette was positioned in the opposite orientation to the original hU6-gRNA expression cassette to reduce the possibility of recombination (Fig 1A). The additional cassette also contains a BbsI golden gate site at the guide insertion site as per the original cassette. However, unlike the original BbsI site which generates GTTT and GGTG overhangs, the new site generates CGGT and TTTA overhangs (Fig 1B) allowing simultaneous targeted insertion of two annealed oligonucleotides with different complementary overhangs in a one-step digestion-ligation reaction (Fig 1C; see below). We added the extra gRNA cassette to the following Cas9 nuclease vectors: pX330 (no selection marker), pX458 (GFP selection marker) and pX459.V2.0 (puromycin selection marker), and to the following Cas9-nickase vectors: pX335 (no selection marker), pX461 (GFP selection marker) and pX462.V2.0 (puromycin selection marker). Those vectors were named pDG330, pDG458, pDG459, pDG335, pDG461 and pDG462, respectively.

### Efficient generation of custom dual-gRNA vector using a one-step cloning protocol

Having generated the dual-gRNA vectors, we next tested whether we could simultaneously insert two annealed oligonucleotide duplexes in a one-step cloning process. We designed two gRNA oligonucleotide inserts targeting the mouse *Sox1* and *Sox3* genes. These inserts carried BspMI and SacI restriction sites at the original and second hU6-gRNA sites, respectively. Annealed oligonucleotide duplex pairs and pDG459 vector were subjected to a one-step digestion-ligation cycling protocol followed by bacterial transformation (Fig 1C). All 12 colonies analyzed contained vectors with correct assembly based on their RFLP pattern (Fig 1D). Similar results were obtained with other dual-gRNA plasmids (pDG330, pDG335, pDG461 and pDG462) with correct assembly in 21/23 colonies based on RFLP, confirmed by sequencing in 9 samples (data not shown). This demonstrates that our dual-gRNA vector design combined with the one-step cloning protocol can allow easy and efficient generation of CRISPR/Cas9 vectors with dual-gRNA expression cassettes.

### Efficient generation of DSB at two sites using vectors expressing Cas9 nuclease and dual-gRNAs

**Fig 2:**
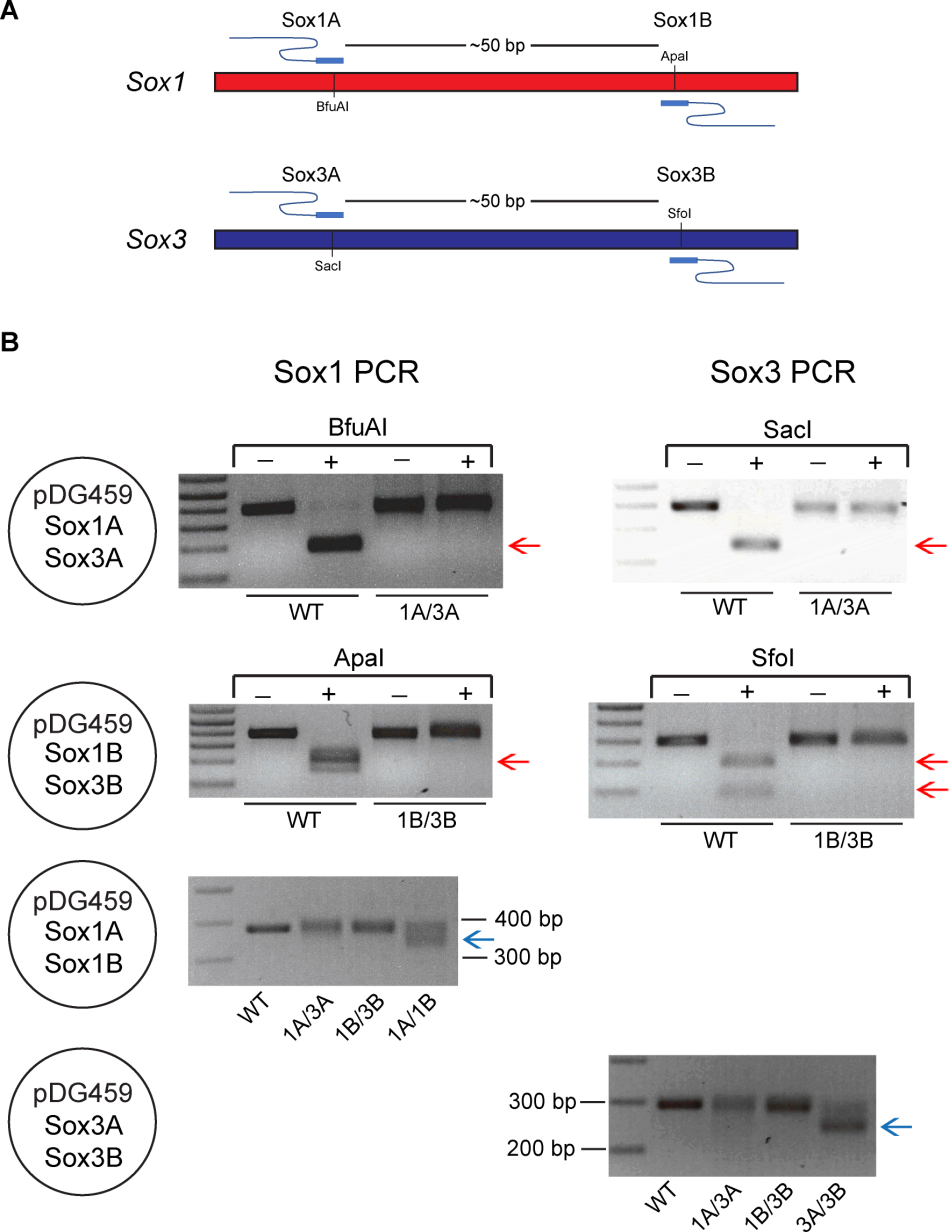
Efficient dual cutting mediated by pDG459 vector. (A) Schematic of gRNA target sites in the *Sox1* and *Sox3* genes. (B) Highly efficient dual cuts induced by vectors derived from pDG459 as indicated by PCR and RFLP analyses. WT products were cut by restriction enzymes resulting in bands indicated by the red arrows. Absence of these bands in dual-gRNA vector-treated samples indicated that the Cas9 nuclease and the gRNAs efficiently induced mutations thus destroying the restrictions sites. Efficient cuts from pDG459 Sox1A/Sox1B and pDG459 Sox3A/Sox3B were indicated by deletion of ~50 bp regions between cuts (blue arrows). Complete figures with more independent samples can be found in S1 Fig.

We next tested whether the dual-gRNA Cas9-nuclease vectors could efficiently induce indels or deletions through simultaneous digestion at two target sites. Four different pDG459 derivatives were initially generated; the first targeted *Sox1* site A and *Sox3* site A (pDG459 Sox1A/Sox3A), the second targeted *Sox1* site B and *Sox3* site B (pDG459 Sox1B/Sox3B), the third targeted *Sox1* site A and *Sox1* site B (pDG459 Sox1A/Sox1B) which are separated by 51 bp and the last targeted *Sox3* site A and *Sox3* site B (pDG459 Sox3A/Sox3B) which are separated by 47 bp (Fig 2A). All target sequences contained restriction sites and hence indel generation at each site could be assayed by RFLP analyses. In addition, efficient digestion by pDG459 Sox1A/Sox1B or pDG459 Sox3A/Sox3B gRNAs should cause a deletion of ~50 bp which can be readily detected by PCR. Each of the four constructs were separately transfected to the mouse ES cells followed by puromycin selection to ensure only transfectants were harvested. *Sox1* and *Sox3* PCRs were performed on Sox1A/Sox3A-treated samples followed by a BfuAI (isoschizomer of BspMI) and SacI RFLP assay to assess indel generation at Sox1A and Sox3A sites, respectively. Both RFLP analyses indicated that pDG459 Sox1A/Sox3A plasmid induced mutations with ~100% efficiency at both Sox1A and Sox3A sites (Fig 2B and S1 Fig). Highly efficient mutagenesis of the Sox1B and Sox3B sites was also detected by ApaI and SfoI RFLP assays in pDG459 Sox1B/Sox3B-trasfected cells (Fig 2B and S1 Fig). We next examined whether deletion of the sequences between the cut sites could be induced by pDG459 Sox1A/Sox1B or Sox3A/Sox3B transfection. PCR products corresponding to deletion alleles were readily generated in pDG459 Sox1A/Sox1B- or Sox3A/Sox3B-treated samples but not in the WT and the unpaired controls upon *Sox1* or *Sox3* PCR (Fig 2B, S1 Fig). Efficient dual nuclease activity was also demonstrated using pDG330- and pDG458-derived constructs (S2 Fig). Together, these data indicate that all-in-one dual-gRNA Cas9 nuclease vectors can facilitate efficient simultaneous cutting at two gRNA target sites.

### Efficient DSBs induced by plasmids expressing Cas9-nickase and dual paired-gRNAs

**Fig 3:**
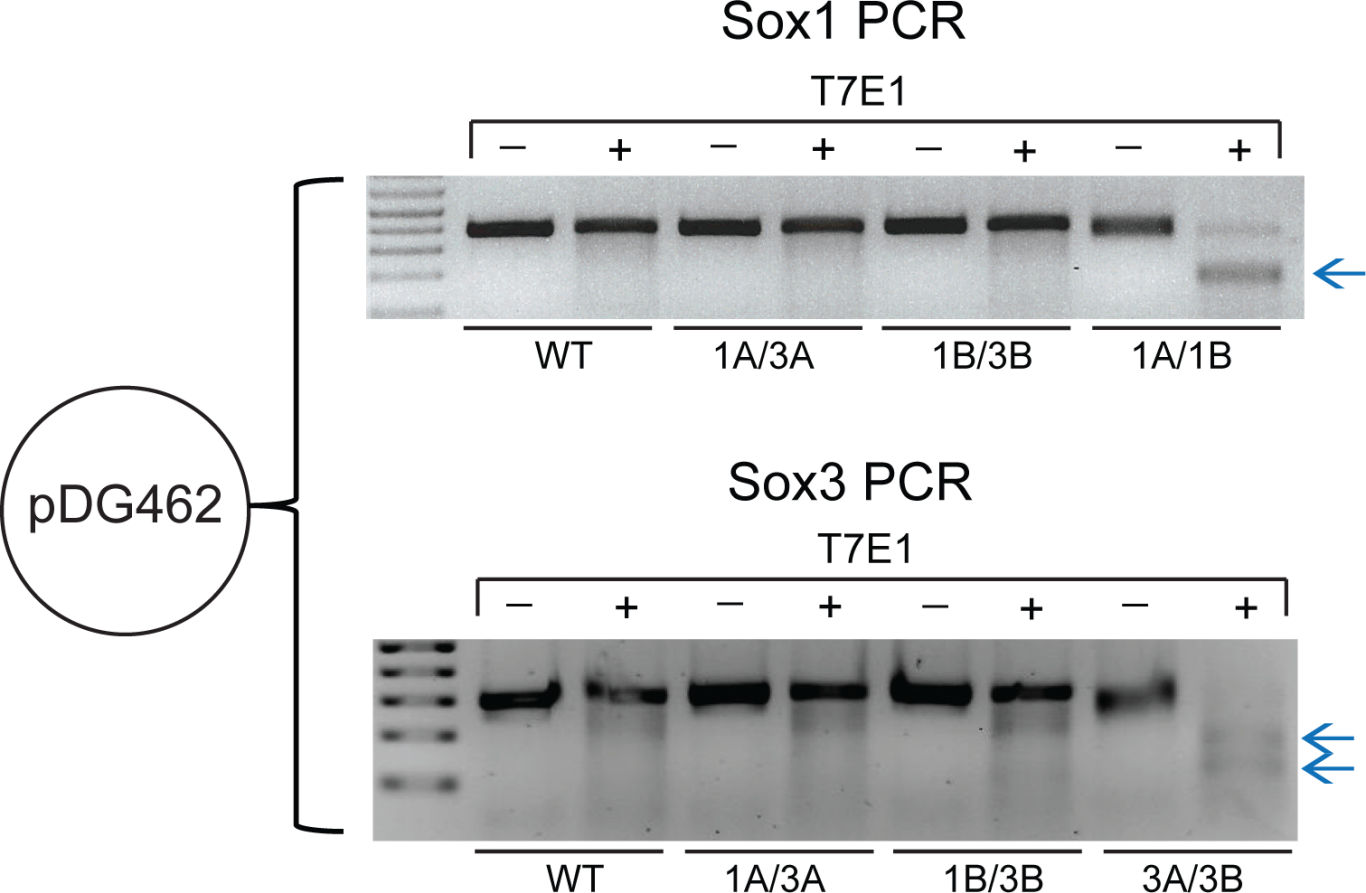
Paired-nickase DSB induction by pDG462. *Sox1* or *Sox3* PCR followed by T7E1 assay was performed on pDG462-transfected samples. Mutations in *Sox1* and *Sox3* were induced by pDG462 Sox1A/Sox1B or pDG462 Sox3A/Sox3B, respectively, as indicated by the digested products after T7E1 treatment (blue arrows). Mutations were not induced by non-paired-nickase control plasmids (pDG462 Sox1A/Sox3A or pDG462 Sox1B/Sox3B). Complete figures with more independent samples can be found in S3 Fig.

Expression of Cas9-nickase with a single gRNA results in a ssDNA break that is typically repaired without causing a mutation. In contrast, expression of Cas9-nickase and two gRNAs targeting closely spaced sites on opposite DNA strands will generate a staggered DSB, repair of which results in indel mutations [10, 11]. We next tested the dual-gRNA Cas9-nickase vectors to assess whether they could efficiently induce DSBs via expression of gRNA pairs. We generated pDG462 derivatives targeting Sox1A/Sox1B and Sox3A/Sox3B which have the requisite orientation and spacing to permit mutagenesis by paired-nickase activity (Fig 2A). As negative controls, we also generated pDG462 targeting Sox1A/Sox3A and Sox1B/Sox3B which are not paired therefore should not generate indel mutations. Vectors were transfected to mouse ES cells followed by puromycin selection. T7E1 heteroduplex assays revealed that pDG462 Sox1A/Sox1B and Sox3A/Sox3B efficiently generated mutations at *Sox1* and *Sox3*, respectively (Fig 3 and S3 Fig). In contrast, there was no evidence of mutations after transfection of the non-paired control plasmids (Fig 3 and S3 Fig). Efficient mutation of *Sox3* was also achieved using dual-gRNA nickase vectors pDG335 and pDG461 expressing Sox3A/Sox3B (S4 Fig). Together, these data demonstrate efficient targeted mutagenesis using dual-gRNA paired-nickase vectors.

## Discussion

Plasmids from the Zhang laboratory have greatly simplified generation of customized gRNA-Cas9/Cas9-nickase expression constructs through utilization of the golden gate cloning strategy. Users only need to anneal a pair of oligonucleotides and ligate them into the vectors via a one-step cloning process, circumventing multiple rounds of PCR and cloning [6, 9]. We modified available plasmids to allow simultaneous insertion of two oligonucleotide duplex inserts using the simple one-step cloning method. These modified vectors provide a user-friendly and cost-effective system to perform experiments that require simultaneous expression of two gRNAs. Additionally, we have shown that both gRNA cassettes are active and induce mutations with high efficiency at both target sites when combined with reliable transfection and selection methods.

Other recent studies have also generated CRISPR/Cas9 vectors that are able to express dual-gRNAs simultaneously, most of which also take advantage of golden gate cloning. However, unlike the dual-gRNA vectors described herein, these require multiple rounds of cloning and/or PCR [12–15]. Additionally, the strategy to express dual-gRNA as a polycistronic transcript that is split by Csy4 RNA polymerase [16] has been shown to have low efficiency [17]. Furthermore, our dual-gRNA vectors are available with Cas9 nuclease or nickase, and with or without selection markers, and can therefore be utilized in a broad range of experimental contexts. Vectors from other studies, although more complicated, are useful when conducting experiments requiring more than 2 gRNAs since those vectors can bear up to 7 gRNAs in a single vector [13, 14].

Our one-step cloning strategy could be applied to generate multiple gRNAs by adding more hU6-gRNA cassettes. To do so, the BbsI sites of the new cassettes would need to be modified to produce different unique overhangs upon digestion. This cloning approach could also be combined with other commonly used CRISPR platform variants such as Cpf1, dCas9-Fok1, Cas9-HF, eSpCas9, and other Cas9 orthologs or mutants that recognize different PAM sequences.

Off-target mutagenesis is one of the most significant issues of CRISPR/Cas9 genome editing [18, 19], particularly for therapeutic applications. The paired-nickase strategy has previously been shown to minimize the off-target effects that are a feature of Cas9 nuclease [20, 21]. We therefore anticipate that the dual-gRNA nickase vectors will be an attractive option for users who require efficient mutagenesis and with maximum specificity.

Efficient dual nuclease cuts are useful for generating targeted large deletions for many purposes such as studying the function of enhancers or long non-coding RNA. In some situations, targeted large deletions are required to delete an exon such as for DMD therapeutics via exon skipping [22–24] or to delete a centromere for chromosome removal [25]. Dual-gRNA Cas9 vectors could also be used for simultaneous KO of two different genes. We also offer our dual-gRNA nuclease vectors for efficient generation of chromosome translocations to model diseases such as Burkitt’s lymphoma or acute myeloid leukemia [12]. Dual DSBs may also aid insertion of flanking loxP sequences for conditional deletion and for insertion of gene swap constructs [26, 27]. Furthermore, these vectors can also be used for injection into mouse zygotes for the generation of mutant mice [28]. Taken together our vectors are a valuable addition to the CRISPR/Cas9 toolbox and should be useful for many CRISPR/Cas9-based applications.

## Materials and methods

### Plasmid and gRNA design

Plasmids pX330, pX335, pX458, pX459.V2.0, pX461 and pX462.V2.0 were gifts from Feng Zhang (Addgene plasmid 42230, 42335, 48138, 62988, 48140 and 62987, respectively) [6, 9]. The Cas9 or Cas9-nickase of those plasmids are derived from *Streptococcus pyogenes* Cas9 which recognizes NGG PAM sequences. The BbsI sequences from pX330 were replaced with the second version of BbsI sequences (see Fig 1B). The hU6-gRNA region was then amplified using primers containing NotI sites. PCR products were then ligated to original plasmids at the NotI site. Guide sequences targeting Sox1A, Sox1B, Sox3A and Sox3B were 5’-GCCGCCGGGCGAGTGCAGGT-3’, 5’-GCCCACGAACCTCTCGGGCC-3’, 5’-GCTGACCCACATCTGAGCTC-3’ and 5’-GACCGCAGTCCCGGCGCCC-3’, respectively, which were designed using online CRISPR design tool http://crispr.mit.edu/. The modified plasmids have been submitted to Addgene with plasmid reference number #100898–100993.

### One step cloning for the generation of customized dual-gRNA plasmid

Forward and reverse oligonucleotides containing the guide sequences for Sox1A, Sox1B, Sox3A and Sox3B with appropriate overhangs (Table 1) were phosphorylated and annealed by mixing 100 pmol of each pair and 0.5 μL T4 PNK (NEB) then incubated at 37°C for 30 minutes, 95°C for 5 minutes and slowly ramped to RT. Annealed oligonucleotides were diluted 1 in 125. Pairs of oligonucleotide duplexes were ligated into the empty vectors in a one-step digestion ligation reaction by mixing the diluted duplex oligonucleotide pairs (1 μL each) with 100 ng empty vector, 100 μmol of DTT, 10 μmol of ATP, 1 μL of BbsI (NEB), 0.5 μL of T4 ligase (NEB) and NEB-2 buffer in 20 μL of reaction. The mixture was placed in a thermocycler and cycled 6 times at 37°C for 5 minutes and 16°C for 5 minutes before bacterial transformation. Plasmids were prepared using miniprep kit (Qiagen) or PureLink® HiPure Plasmid Midiprep Kit (Life Technologies). Correct insertion of oligonucleotide duplexes into the vectors was confirmed by Sanger sequencing using the following primers: GGTTTCGCCACCTCTGACTTG (first insert) and TGCATCGCATTGTCTGAGTAGG (second insert). It is recommended to digest the vectors using BbsI before sequencing as correct insertion should remove the BbsI sites.

**Table 1:** List of oligos used to generate the dual-gRNA targeting plasmids.

Target	Oligo pair 1 (5’-3’)	Oligo pair 2 (5’-3’)
Sox1A/Sox3A	CACCGCCGCCGGGCGAGTGCAGGT	ACCGCTGACCCACATCTGAGCTCGT
	AAACACCTGCACTCGCCCGGCGGC	TAAAACGAGCTCAGATGTGGGTCAG
Sox1B/Sox3B	CACCGCCCACGAACCTCTCGGGCC	ACCGACCGCAGTCCCGGCGCCCGT
	AAACGGCCCGAGAGGTTCGTGGGC	TAAAACGGGCGCCGGGACTGCGGT
Sox1A/Sox1B	CACCGCCGCCGGGCGAGTGCAGGT	ACCGCCCACGAACCTCTCGGGCCGT
	AAACACCTGCACTCGCCCGGCGGC	TAAAACGGCCCGAGAGGTTCGTGGG
Sox3A/Sox3B	CACCGCTGACCCACATCTGAGCTC	ACCGACCGCAGTCCCGGCGCCCGT
	AAACGAGCTCAGATGTGGGTCAGC	TAAAACGGGCGCCGGGACTGCGGT

Grey highlights indicate the sequence of the guides

### Cell culture and transfection

R1 mouse embryonic stem cells from Andras Nagy’s laboratory (Established from a male blastocyst hybrid of two 129 substrains (129X1/SvJ and 129S1/SV-+^p^+^Tyr-c^ Kitl ^Sl-J^/+)) were used for all experiments. Cells were cultured in 15% FCS/DMEM supplemented with LIF, 3 μM CHIR99021 (Sigma), 1 μM PD0325901 (Sigma), 2 mM Glutamax (Gibco), 100 μM non-essential amino acids (Gibco) and 100 μM 2-mercaptoethanol (Sigma). One million ES cells were nucleofected with 3 μg of plasmid DNA using the Neon™ Transfection System 100 μL Kit (Life technologies) at 1400 V, 10 ms and 3 pulses according to the manufacturer’s protocol. For transfection of pDG459 and pDG462, puromycin selection (2 μg/mL was initiated 24 hours post transfection for 48 hours. GFP FACS was performed on cells transfected with pDG458 and pDG461 48 hours post transfection. Surviving cells were cultured for 4–7 days without selection before harvesting. Cells transfected with plasmid pDG330 and pDG335 did not undergo any selection.

### DNA extraction, PCR, RFLP and T7E1 assay

Genomic DNA was extracted from 1–2 million cells using High Pure PCR Template Preparation Kit (Roche) according to the manufacturer’s instructions. *Sox1* PCR was performed using primers F: 5’-CCCTTCTCTCCGCTAGGC-3’ and R: 5’-GTTGTGCATCTTGGGGTTTT-3’. *Sox3* PCR used primers F: 5’-CAGCATGTACCTGCCACCT-3’ and R: 5’-ACAAAACCCCGACAGTTACG-3’. RFLP or T7E1 assay was performed by mixing 5 μL of PCR products (without purification) with the restriction enzymes or T7E1 enzyme (NEB) in a total volume of 20 μL and incubated for 1 hour at the suggested optimal temperatures. Prior to T7E1 assay, PCR products were slowly re-annealed to form heteroduplex products by heating the PCR products at 95°C for 5 minutes and slowly ramped down to room temperature.

## Supporting information

S1 Fig Efficient dual cutting mediated by pDG459 vector. Extended figures of Fig 2B with more independent samples. (A) BfuAI and SacI RFLP analyses indicated efficient dual cuts from pDG459 Sox1A/Sox3A. (B) ApaI and SfoI RFLP analyses indicated efficient dual cuts from pDG459 Sox1B/Sox3B. WT products after digestions (red arrows) were absent in pDG459-treated samples. (C) Large deletions were induced in the *Sox1* region in pDG459 Sox1A/Sox1B-treated samples. (D) Large deletions were induced in the *Sox3* region in pDG459 Sox3A/Sox3B-treated samples. Large deletion fragments are indicated with blue arrows. Each sample came from independent transfection (n ≥ 3).
(TIF)

S2 Fig Mutation inductions mediated by vectors pDG330 and pDG458. (A) Transfection of pDG330 Sox1A/Sox3A into mouse ES cells induced mutations at both targets which were indicated by smaller fragments after T7E1 assay (blue arrows). (B) BfuAI and SacI RFLP were used to assess the mutation induction in Sox1A and Sox3A sites, respectively, after treatment of pDG458 Sox1A/Sox3A followed by GFP FACS enrichment. Presence of WT products produced smaller bands after restriction digestions (red arrows) which were absent in pDG458 Sox1A/Sox3A-treated samples. Each sample came from independent transfection.
(TIF)

S3 Fig Paired-nickase DSB induction by pDG462. Extended figures of Fig 3 with more independent samples. Smaller bands produced after T7E1 digestion (blue arrows) indicated presence of mutation in samples treated with paired-nickase pDG462 Sox1A/Sox1B (A) or Sox3A/Sox3B (B). Each sample came from independent transfections.
(TIF)

S4 Fig Paired-nickase-mediated mutation inductions by pDG335 and pDG461 vectors. T7E1 assay showed that expression of paired-nickase gRNAs Sox3A/Sox3B from pDG335 (A) or pDG461 (B) induced mutations in the *Sox3* locus as indicated by the presence of cut products (blue arrows). Each sample came from independent transfections.
(TIF)
