# Supplementary figures and images for: Versatile single-step-assembly CRISPR/Cas9 vectors for dual gRNA expression
Source: PLoS One. 2017 Dec 6;12(12):e0187236. doi: 10.1371/journal.pone.0187236 (PMC5718404; doi:10.1371/journal.pone.0187236)

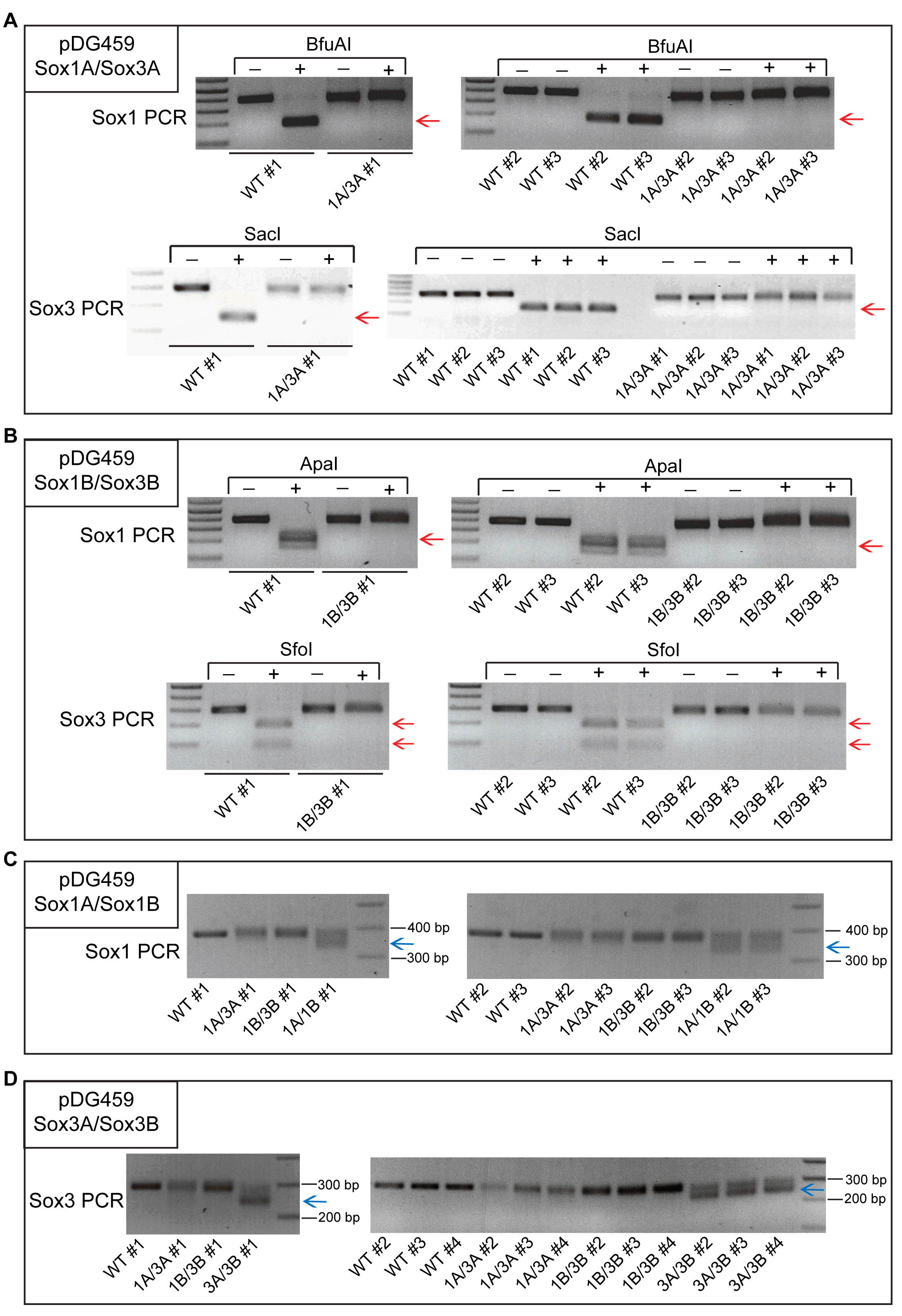

Supplement: S1 Fig — Extended figures of Fig 2B with more independent samples. (A) BfuAI and SacI RFLP analyses indicated efficient dual cuts from pDG459 Sox1A/Sox3A. (B) ApaI and SfoI RFLP analyses indicated efficient dual cuts from pDG459 Sox1B/Sox3B. WT products after digestions (red arrows) were absent in pDG459-treated samples. (C) Large deletions were induced in the Sox1 region in pDG459 Sox1A/Sox1B-treated samples. (D) Large deletions were induced in the Sox3 region in pDG459 Sox3A/Sox3B-treated samples. Large deletion fragments are indicated with blue arrows. Each sample came from independent transfection (n ≥ 3). (TIF) [file pone.0187236.s001.tif]

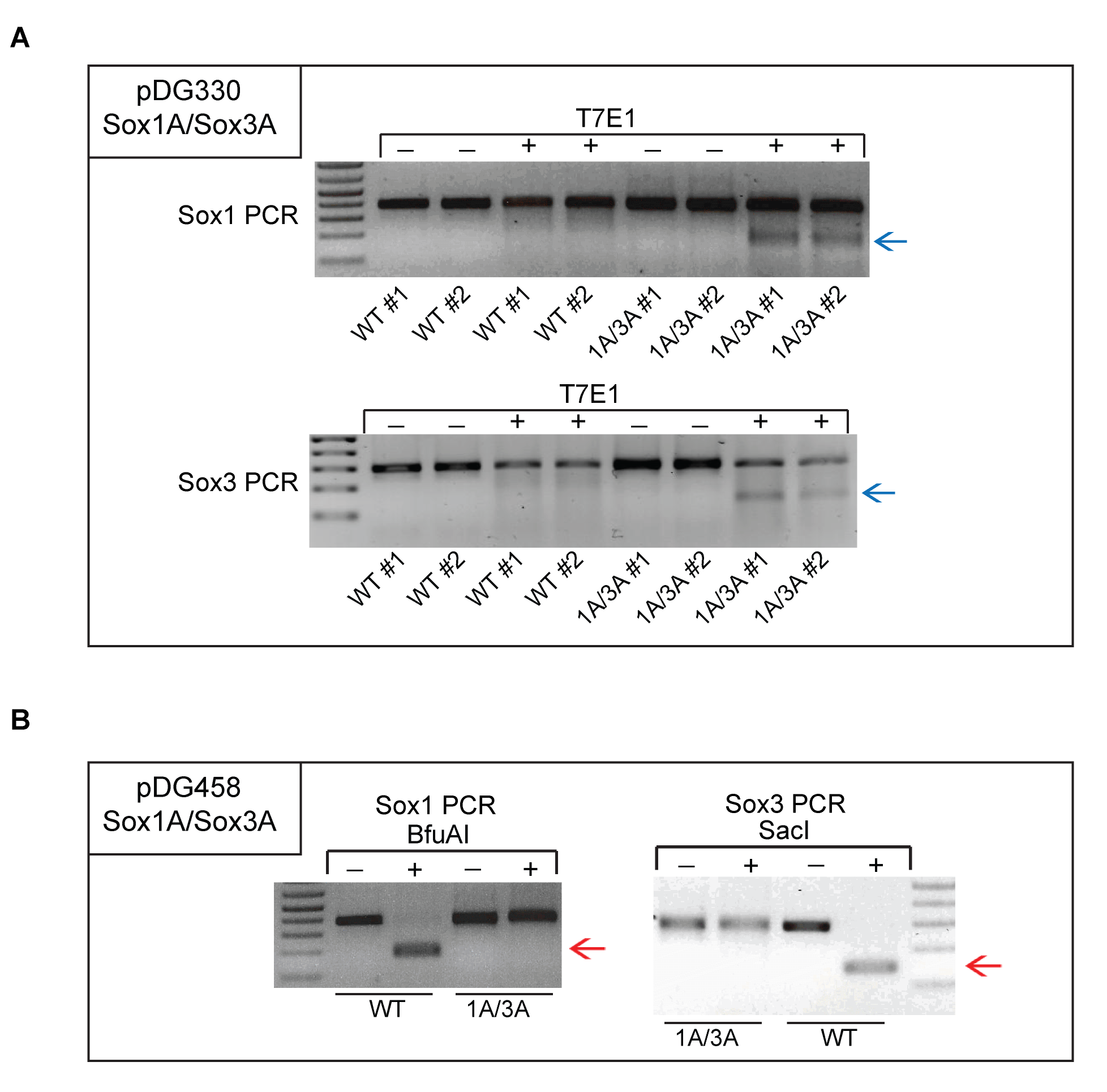

Supplement: S2 Fig — (A) Transfection of pDG330 Sox1A/Sox3A into mouse ES cells induced mutations at both targets which were indicated by smaller fragments after T7E1 assay (blue arrows). (B) BfuAI and SacI RFLP were used to assess the mutation induction in Sox1A and Sox3A sites, respectively, after treatment of pDG458 Sox1A/Sox3A followed by GFP FACS enrichment. Presence of WT products produced smaller bands after restriction digestions (red arrows) which were absent in pDG458 Sox1A/Sox3A-treated samples. Each sample came from independent transfection. (TIF) [file pone.0187236.s002.tif]

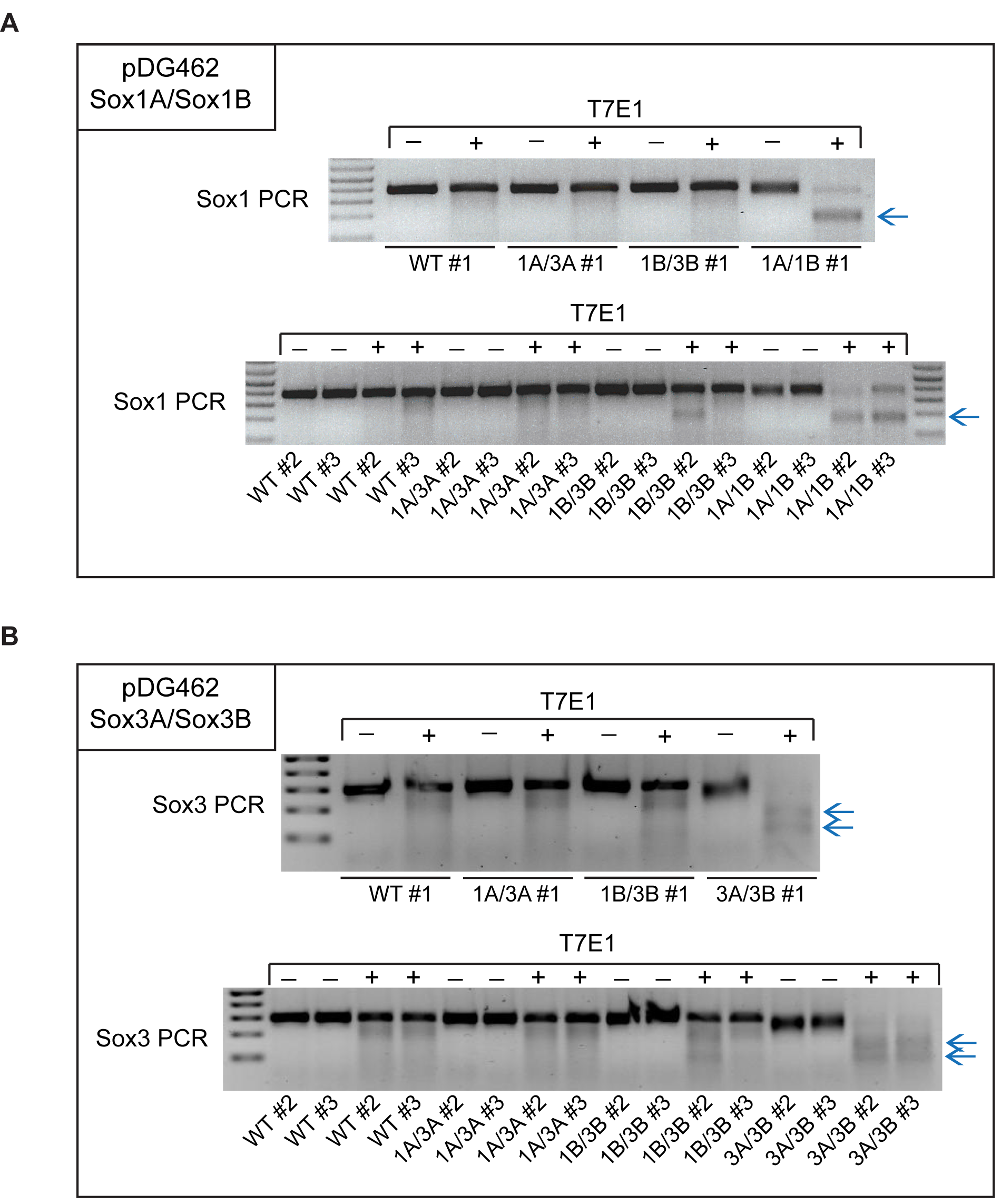

Supplement: S3 Fig — Extended figures of Fig 3 with more independent samples. Smaller bands produced after T7E1 digestion (blue arrows) indicated presence of mutation in samples treated with paired-nickase pDG462 Sox1A/Sox1B (A) or Sox3A/Sox3B (B). Each sample came from independent transfections. (TIF) [file pone.0187236.s003.tif]

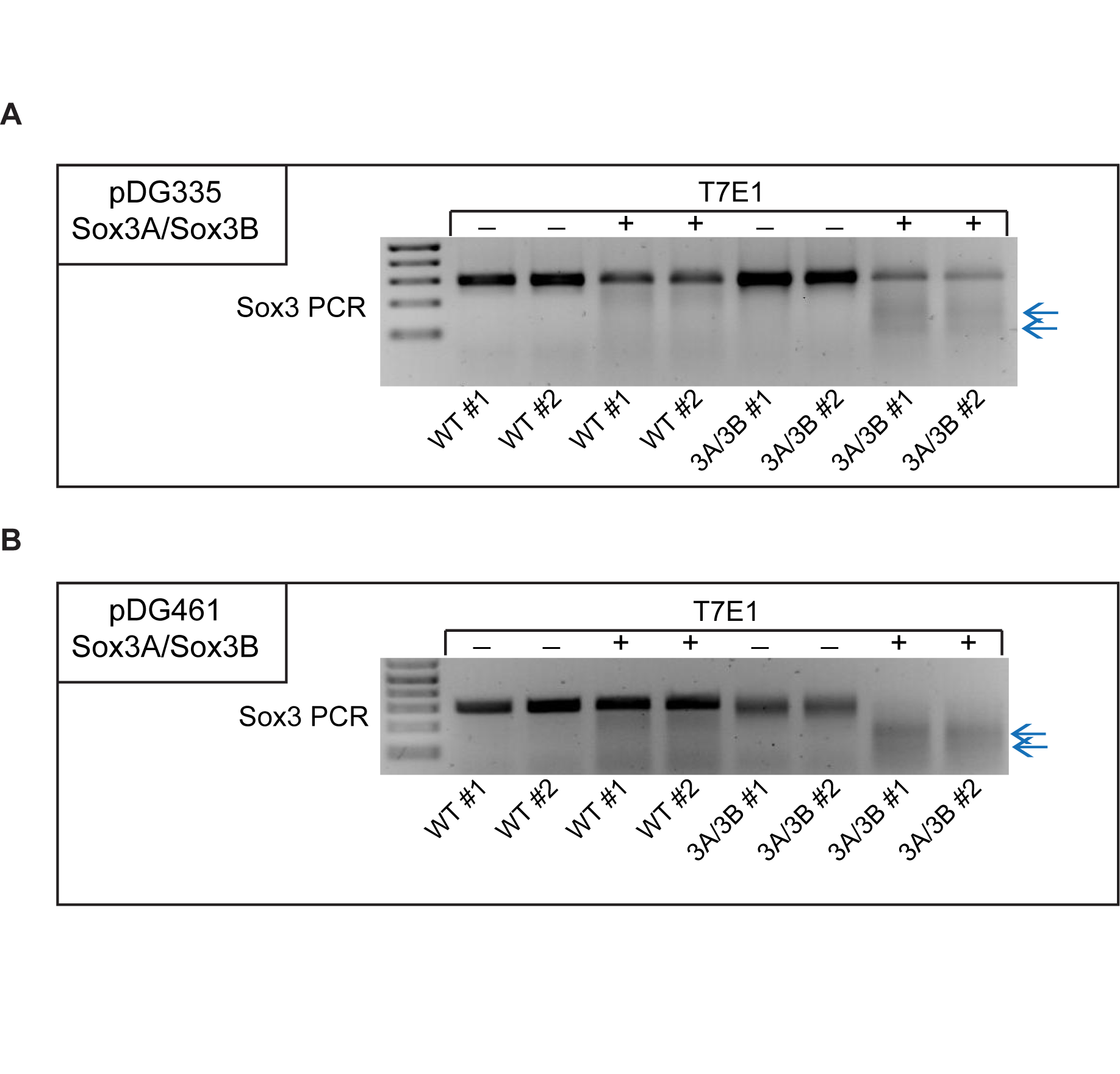

Supplement: S4 Fig — T7E1 assay showed that expression of paired-nickase gRNAs Sox3A/Sox3B from pDG335 (A) or pDG461 (B) induced mutations in the Sox3 locus as indicated by the presence of cut products (blue arrows). Each sample came from independent transfections. (TIF) [file pone.0187236.s004.tif]
